# Patient-Specific Instrument Guided Double Chevron-Cut Distal Femur Osteotomy

**DOI:** 10.3390/jpm11100959

**Published:** 2021-09-26

**Authors:** Yen-Chun Huang, Kuan-Jung Chen, Kuan-Yu Lin, Oscar Kuang-Sheng Lee, Jesse Chieh-Szu Yang

**Affiliations:** 1 School of Medicine, National Yang Ming Chiao Tung University, Taipei 112, Taiwan; fu6294613@gmail.com (Y.-C.H.); ronald96016@gmail.com (K.-J.C.); 2 Department of Orthopedics and Traumatology, Taipei Veterans General Hospital, Taipei 112, Taiwan; oscarlee9203@gmail.com; 3 Department of Orthopedics, Kaohsiung Veterans General Hospital, Kaohsiung 813414, Taiwan; johnkyl@yahoo.com; 4 Department of Orthopedics, China Medical University Hospital, Taichung 114, Taiwan

**Keywords:** femoral, osteotomy, 3D-printed, patient-specific, cutting-guide

## Abstract

The risk of non-union and prolonged periods of protected weight-bearing still remain unsolved issues after distal femur osteotomy (DFO). To improve the stability, we developed the double chevron-cut technique, which is a modified medial closing-wedge DFO guided by a patient-specific instrument. The purpose of this study was to investigate the feasibility and outcome of this operative approach. Twenty-five knees in twenty-three consecutive patients with genu valgum and lateral compartment osteoarthritis that received double chevron-cut DFO were included. The target of correction was 50% on the weight-bearing line (WBL) ratio. Patient-reported outcomes included the Oxford Knee Score (OKS) and the 2011 Knee Society Score (KSS). The mean of the WBL ratio was corrected from 78.7% ± 12.0% to 48.7% ± 2.9% postoperatively. The mean time to full weight bearing was 3.7 ± 1.4 weeks. Union of the osteotomy was achieved at 11.3 ± 2.8 weeks. At a mean follow-up of 17 months, the OKS improved from a mean of 27.6 ± 11.7 to 39.1 ± 7.5 (*p* = 0.03), and the KSS from a mean of 92.1 ± 13.0 to 143.9 ± 10.2 (*p* < 0.001). Three patients developed complications, including one case of peri-implant fracture, one of loss of fixation, and one of non-union. The double chevron-cut DFO followed by immediate weight-bearing as tolerated is effective in treating genu valgum deformity and associated lateral compartment osteoarthritis.

## 1. Introduction

Distal femur osteotomy (DFO) has become increasingly popular in treating patients with genu valgum deformity and associated lateral compartment osteoarthritis. Although genu valgum deformity can also be corrected with high tibial osteotomy, deformity greater than 10° would be better corrected with DFO to avoid iatrogenic joint line obliquity [1,2].

The valgus correction with DFO can be performed with either a medial closing-wedge or lateral open-wedge technique. Although survival rates after the two procedures are similar, medial closing-wedge DFO offers the advantage of native bone-to-bone healing and inherent stability, and thus, an earlier start of weight-bearing activities [3,4]. Nevertheless, the conventional closing-wedge technique is troubled with 3–25% delayed union or non-union, 5% loss of correction, and malrotated correction [5,6,7,8]. The rate of delayed union or non-union hovered around 4–5%, even with the advent of locking plates [8,9].

In order to further improve the inherent stability and increase the contact area of the osteotomized bone, we redesigned the bone cuts of medial closing-wedge DFO as two chevron-shaped cuts. Double chevron-cut closing-wedge osteotomy requires four precise bone cuts converging to a hinge point, while the bone cuts are not necessarily perpendicular to the anteroposterior (AP) view of intraoperative radiographs. It is therefore largely impractical to perform the procedure with a conventional freehand technique, but feasible with the guide of a patient-specific instrument (PSI).

The objective of this study was to assess the feasibility and results of this technique performed on the first 23 consecutive patients, with 25 knees with symptomatic lateral compartment osteoarthritis related to genu valgum.

## 2. Materials and Methods

This retrospective review of prospectively collected data was conducted in accordance with the Declaration of Helsinki [10] and was approved by the ethics committee of the institute. From June 2017 to April 2019, PSI-guided double chevron-cut DFO was performed on a total of 23 consecutive patients and 25 knees. The indication of the surgery was pain from mild to moderate lateral compartment osteoarthritis (grade 1 to 3 on the Kellgren–Lawrence Classification [11]) and valgus deformity from distal femur, indicated by the parameters of weight-bearing line (WBL) ratio >60% and lateral distal femur angle (LDFA) <85°. Cases were not considered for DFO if diagnosed with inflammatory arthritis, ligamentous instability of the knee, osteonecrosis of the lateral femoral condyle, or severe multicompartmental arthritis [12]. All patients were operated on by a single surgeon (J.C.S.Y.). Written informed consent was obtained from all participants.

### 2.1. Preoperative Planning

**Figure 1:**
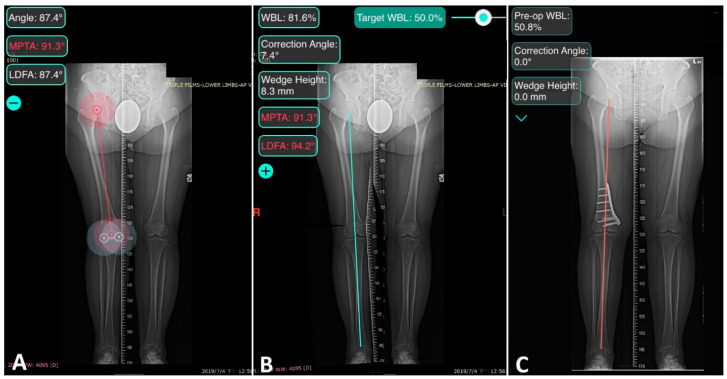
Digital Preoperative Planning. (**A**) The full-length weight-bearing AP radiograph of a 67-year-old female with valgus deformity of the right knee. The femorotibial angle, MPTA, and LDFA were measured automatically in the software. (**B**) A 2D simulation of the osteotomy, setting the target of correction at 50% on the WBL ratio. The correction angle, wedge height, and LDFA after correction were simulated by the software. (**C**) The full-length weight-bearing AP radiograph of the same patient one year after DFO. The WBL ratio was corrected to 50.8%.

The osteotomy was first planned on the full-length weight-bearing AP radiograph with the aid of software (OsteoMaster; 2017 Luo Chu An, Taiwan). The hinge was set on 5 mm medial to the internal cortex of the lateral epicondyle of the femur. The targeted WBL ratio was 50% after correction (Figure 1).

The plan was marked on AP radiographs and 3D reconstructed computer tomography (CT) images were sent to the manufacturer of the PSI and plate system (Anatomic Precision PSI DFO; A-plus Bio., New Taipei City, Taiwan) to construct a virtual 3D surgical planning model, combining 2D planning with 3D-reconstructed CT images by correlating anatomic landmarks. The model was then used to design PSI.

**Figure 2:**
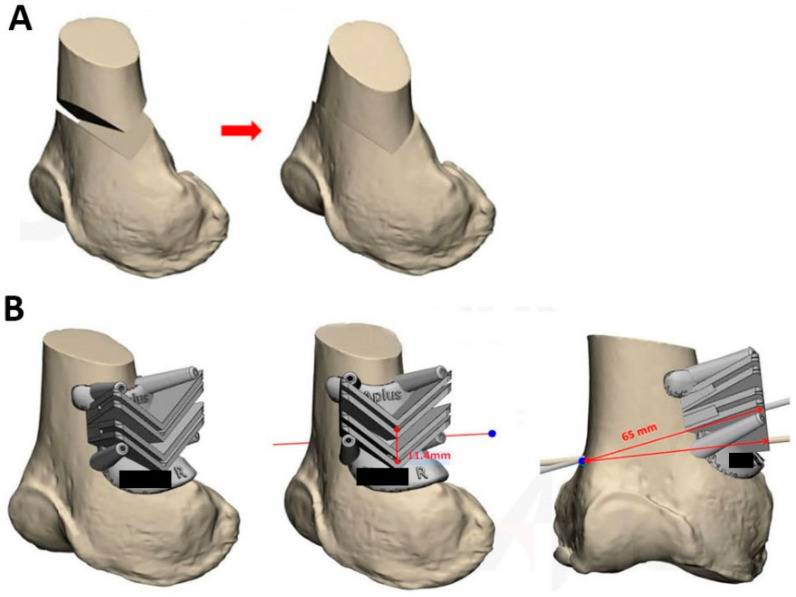
Digital 3D Model Simulation of double chevron-cut DFO. (**A**) The virtual 3D model of the patient in Figure 1. The osteotomy and correction after closing-wedge DFO were simulated. (**B**) A 3D simulation of the 3D-printed cutting jig. The wedge thickness, correction angle, and depth of bone cut were calculated based on the virtual 3D model and printed on the guide as a reminder during the surgery.

A commercialized PSI system is composed of a patient-specific cutting guide and an associated locking plate system. The guide is a single-use, disposable, 3D-printed polyamide cutting jig for the removal of a precise chevron-shaped bone wedge. The guide includes six holes for K-wires and two chevron cutting slots shaped with a mitered angle at 110°, the axis parallel to the medullary canal, and the apex pointing distally (Figure 2). The thickness of the wedge (the distance between two chevron cuts), angle of correction, and depth of the bone cuts were calculated in the virtual 3D model. The parameters of cutting were printed on the guide as a reminder during the surgery.

### 2.2. Surgical Technique

**Figure 3:**
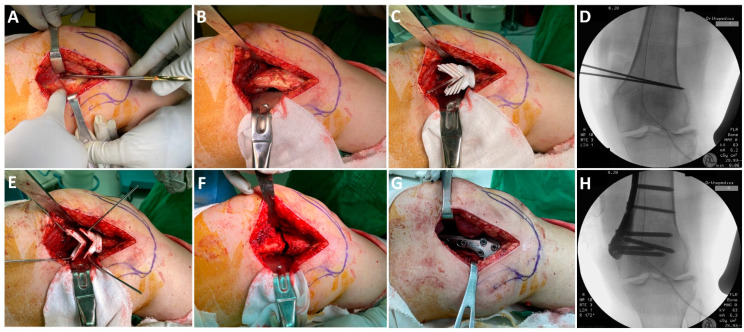
The surgical approach and the intraoperative radiographs. (**A**) A standard medial approach to the distal femur was used, utilizing the plane between the vastus medialis and adductors (distal is to the right and proximal is to the left). (**B**) The periosteum was elevated from the target femoral surface. The posterior femoral structure was retracted with a Bennett retractor in protection of the neurovascular bundle. (**C**) The customized cutting jig was placed onto the medial cortex, identifying the ideal spot with an anatomical fit of the guide. Two orientation K-wires were then inserted through the K-wire holes at the apexes of the chevron cut. (**D**) Positioning of the orientation K-wires was confirmed with a C-arm. (**E**) Four K-wires were inserted, fixing the guide onto the bone. Osteotomy was performed with a graduated oscillating saw. (**F**) The cutting guide and the wedge were removed after the osteotomy. (**G**) The osteotomy was then closed with gentle manual bending and fixed with the associated locking plate system. (**H**) The final position of the plate was confirmed with a C-arm. An alignment rod was not used.

Spinal anesthesia was used in all patients. The patients were placed in a supine position, and a standard medial approach to the distal femur was used [13]. The periosteum was elevated from the target femoral surface. The procedure of the osteotomy is shown in Figure 3. The customized cutting jig was placed onto the medial cortex, identifying the unique spot with an anatomical fit to the guide. Two K-wires were inserted through the K-wire holes at the apexes of the chevron cut to mark the orientation of the guide; the positioning and the proper 5 mm hinge were confirmed with a C-arm. Four additional K-wires were inserted to fix the guide on the bone, followed by removal of the orientation K-wires. Osteotomy was performed with a graduated oscillating saw moved along the cutting slot. The cutting guide was removed after the osteotomy. The bone wedge was removed and osteotomy was then closed with gentle manual bending and fixed with the associated locking plate system. An alignment rod was not used.

### 2.3. Postoperative Care

Oral analgesics and intravenous morphine for breakthrough pain were administered according to a multimodal pain management protocol [14]. Weight-bearing as tolerated was allowed immediately postoperatively. The use of a walker was suggested initially. The patients were evaluated at the out-patient clinic every two weeks until full weight-bearing was achieved.

Standard AP and lateral radiographs were taken postoperatively and every 4 weeks until union of the osteotomy was evident. Full-length weight-bearing AP radiographs were taken preoperatively, two days postoperatively, and on clinical follow-ups every 3 months until consolidation of the osteotomy, and then every year.

### 2.4. Data Collection and Radiographic Assessment

Baseline demographics and operative details were collected from the medical charts, and included: age, sex, body mass index, smoking status, operating time, the number of intra-operative radiographs, preoperative hemoglobin (Hb) level, the Hb level on postoperative day 1, the time to full weight bearing, and complications.

Preoperative radiographic assessments were reviewed to assess Kellgren–Lawrence grade [11]. Parameters of alignment, including LDFA, hip–knee–ankle angle (HKA), and WBL ratio, were measured by software (OsteoMaster; 2017 Luo Chu An, Taiwan) on full-length weight-bearing radiographs taken preoperatively, two days postoperatively, and on the final follow-up. The outlier was defined as those with a difference of more than 10% from the targeted 50% WBL ratio [15]. The union of the osteotomy was identified clinically by the absence of pain and radiographically by diminished osteotomy gap.

The clinical results were evaluated by assessing the patient-reported outcomes before surgery and at the final follow-up visit: Oxford Knee Score (OKS) [16], which has a maximum score of 48 points (a 12-item questionnaire about how an individual’s activities of daily living have been affected by pain; a higher score indicates less pain and better function), and the 2011 Knee Society Score (KSS, a 4-item questionnaire with a maximum score of 180 points, consisting of the patient’s symptoms, satisfaction, expectations, and function) [17]. The active range of motion (ROM) of the knee joint was measured with a goniometer.

All clinical assessments were performed by one of us (K.J.C) at the yearly follow-up examinations, independent of the operating surgeons.

### 2.5. Statistics

Data were analyzed with SPSS (IBM SPSS Statistics for Windows, version 22.0. Armonk, NY, USA). The paired-sample t-test and Wilcoxon signed-rank test were used to compare preoperative and postoperative parameters of alignment and scores. All data were tested for normality. The paired *t*-test was used for normally distributed data (i.e., HKA and WBL ratio), while the Wilcoxon signed-rank test was used for the rest.

## 3. Results

The study evaluated 25 knees from 23 patients with mean age 65 ± 8 years consisting of 78.2% female. The mean follow-up period was 17 ± 8 months (range, 12–37 months). The baseline characteristics are shown in Table 1.

The mean operative time was 58.8 ± 18.3 min. On average, 6.2 ± 1.3 radiographs were taken intraoperatively. During the operation, the mean Hb level decreased from 13.5 ± 1.3 g/dL to 12.1 ± 1.2 g/dL (*p* = 0.001); no patient received blood transfusion (Table 2).

The mean time to full weight bearing was 3.7 ± 1.4 weeks (range 2–8). Union of the osteotomy was achieved in 11.3 ± 2.8 weeks. All patient-reported outcome showed significant improvements. The OKS increased from 27.6 ± 11.7 preoperatively to 39.1 ± 7.5 on the final follow-up (*p* = 0.03). The KSS improved from 92.1 ± 13.0 preoperatively to 143.9 ± 10.2 on the final follow-up (*p* < 0.001). The final active ROM was similar, from 131° ± 4° to 128° ± 10° (*p* = 0.18) (Table 3).

### 3.1. Radiological Results

**Figure 4:**
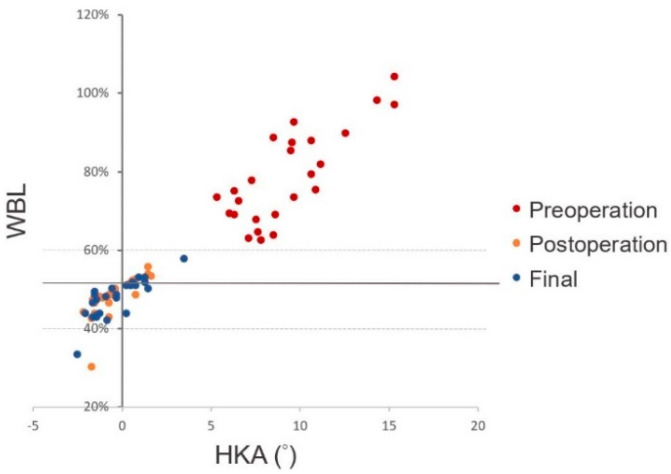
The scatter plot of HKA and WBL ratios of all patients measured preoperatively, 2 days postoperatively, and at the final follow-up. Only one knee (4%) at 2 days postoperatively, and in the final radiographs, fell in the defined range (WBL 50% ± 10%) of outliers of correction.

The WBL ratios were corrected from 78.7% ± 12.0% preoperatively to 48.7% ± 2.9% two days postoperatively (*p* < 0.001). Meanwhile, the average HKA was restored significantly from 9.3° ± 2.8° valgus to 0.5° ± 1.1° varus (*p* < 0.001). The LDFA changed significantly from 83.6° ± 1.9° to 91.4° ± 3.5° (*p* < 0.001). The WBL ratio, HKA, and LDFA remained consistent, comparing two days postoperatively to the final radiographs (*p* = 0.44, *p* = 0.58, and *p* = 0.32, respectively). Only one knee (4%) at two days postoperatively, and in the final radiographs, fell in the defined range of correction outliers (Table 4, Figure 4).

### 3.2. Postoperative Complications

Three cases of complications were identified. One case (4%) of loss of fixation from a fall 6 weeks postoperatively. The patient received revision medial plating using the same implants. One patient (4%) experienced peri-implant fracture superior to the implants 5 weeks postoperatively due to a traffic accident. The patient received removal of the plate followed by fixation with a retrograde interlocking nail. Non-union happened in one knee (4%) of a smoker who received simultaneous bilateral DFO. The patient thereby received bone grafting and additional plating at the lateral side 7 months after the initial DFO surgery.

## 4. Discussion

The present study demonstrated that patients receiving PSI-guided double chevron-cut DFO, followed by immediate weight-bearing as tolerated, achieved full weight bearing at a mean of 3.3 weeks and union after 11.3 weeks. Ninety-two percent of the treated patients achieved targeted correction. The KSS and OKS scores significantly improved after the treatment.

DFO is a widely accepted treatment option for lateral unicompartmental arthritis with valgus deformity. It relieves pain and restores function of the affected knee [6,18,19]. High rates of satisfactory results and high cumulative survival ranging from 78% to 89% was found across studies at 10 years, with conversion to total knee arthroplasty as the endpoint. The survival rate generally declined to 45–71% at 15 years, and 24% at 20 years [18,20,21,22].

The medial closing-wedge and lateral open-wedge DFO provide similar union, survival, and complication rates [3,4,12]. However, a 4–12-week period of non-weight bearing is typically required after lateral open-wedge DFO [4], while immediate partial weight-bearing for 6–8 weeks is allowed after medial closing-wedge DFO in the series with locking plate fixation [8,9,23].

Although closing-wedge osteotomy theoretically offers the advantage of native bone-to-bone healing and inherent stability, conventional techniques still face a profile of complications. Studies in the 1990s used blade plates for closing-wedge DFO. A 3–25% rate of delayed union or non-union, a 5% rate of loss of correction, and malrotated correction have been mentioned in several studies [5,6,7]. Partial weight-bearing started from 6–8 weeks after the surgery, and union of the osteotomy required 2–9 months [4]. In the 2010s, studies reported results of lock plating still revealed a 4–5% rate of delayed union or non-union [8,9].

**Figure 5:**
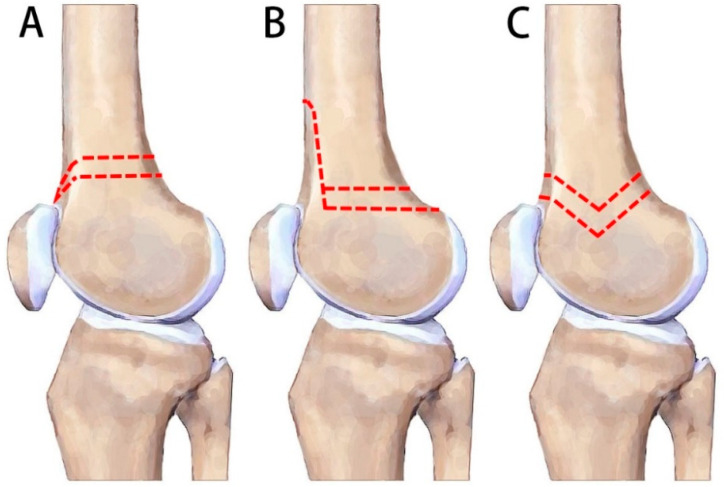
A comparison of DFO techniques. (**A**) Uniplanar technique. (**B**) The “three-cut” biplanar technique. (**C**) The “four-cut” double chevron-cut technique. Note that the distal-most possible site of the biplanar is farther than in the uniplanar technique, since it avoids the femoral trochlea. The double chevron-cut technique may be performed even more distally, since it also avoids the posterior condyle of the femur.

To improve the inherent stability of osteotomy, biplanar osteotomy has been proposed [9]. Van der Woude et al. demonstrated that biplanar technique shortens bone healing time for 2 months in comparison with single plane technique (4 months vs. 6 months, respectively) [23]. Biomechanical studies using sawbone models reported that the femoral contact surface area of biplanar osteotomy increased more than two-fold in comparison to the uniplanar technique [24]. However, another study, although corroborating the improved axial stability and stiffness in biplanar technique, found biplanar osteotomy resulted in less torsional stability than the single-plane technique [25]. This phenomenon may be explained by the observation in a clinical study by Nha et al., which found that the anterior flange of osteotomies was prone to fracture in patients with femurs small in diameter [26]. Without a solid anterior flange, the contact area of the biplanar was merely similar to that of single-plane osteotomies (Figure 5A,B) [24].

Double chevron-cut osteotomy was developed in order to address the weaknesses associated with the anterior flange in the typical biplanar method. The chevron shape has been shown to be one of the biomechanically superior constructs, and is utilized in various sites of the body, such as calcaneus, the first metatarsal, and subtrochanteric femur [27,28,29]. In comparison to the regular biplanar technique, the double chevron-cut technique avoids the breakage of posterior condyle and femoral trochlea. Therefore, it bears the potential to place the osteotomy site more distally into the area of cancellous bone in the femur, a region with better healing potential (Figure 5C).

In our study, the patients were allowed immediate weight-bearing as tolerated, followed by gradual recovery to full weight bearing in 3.7 ± 1.4 weeks, as opposed to the typical 6–8 weeks of partial weight-bearing in most series of closing-wedge DFO [4]. In other words, the patients here restored full weight-bearing capability within as short as half the time of most reported cases took with partial weight bearing. Meanwhile, the time to union (11.3 weeks) and the rate of non-union or loss of fixation (both 4%) were similar to those in published studies. These results are especially promising, considering the cohort in the current study was older (average 65 years) than the ones in most reported studies. In the only patient where non-union was found, the factors likely contributed to the non-union were the smoking habit and simultaneous bilateral surgery.

Double chevron-cut closing-wedge osteotomy requires a customized cutting jig (PSI guidance) since the procedures demand high precision. It asks for four accurate bone cuts converging to a single hinge point; none of the bone cuts are necessarily perpendicular to the AP view of intraoperative radiographs, unlike most typical DFO procedures. Conventional freehand closing-wedge technique, although already known to be rigorous, is not compatible with the demands of an even smaller margin of error here, as it is highly reliant on preoperative planning and accuracy of resection [3,4,30]. The design of the PSI system here also allowed easy identification of osteotomy spot by fitting the guide with unique shape to the specific site on the femur. The system enables minute control over the orientation and the cuts adhering to preoperative plans. Several studies also had demonstrated PSI can achieve better precision of correction, shorter surgical time, and less radiographic exposure in comparison with conventional DFO techniques [31,32,33]. PSI had been utilized in both open-wedge (a single bone cut) and close wedge technique (two bone cuts) DFO. Our current study here attempted the use of PSI in a chevron-cut closing-wedge technique (four bone cuts). The operative time of the technique takes on average 58.8 min and 6.2 intraoperative radiographs. The deviation from the plan was 1.3% in the WBL ratio. The results were similar to the PSI groups of previous studies [31,32,33].

The PSI cutting guide typically takes three days to be manufactured upon receiving the plan on radiograph and 3D reconstructed CT images. The cost of the PSI and plate system is 5385 USD, in comparison with 2653 USD for a similar locking plate system without PSI (TomoFix Medial Distal Femur Plate, DePuy Synthes, Raynham, MA, USA). The increased cost and waiting time are potential limitations in comparison with the classical technique.

There are some limitations in the present study. Firstly, the number of patients included was relatively small, and it lacked a control group. Secondly, the cohort consisted of the first group of patients who were operated on with this new technique, and it is therefore likely that there was a learning curve. Thirdly, union of the fracture was evaluated clinically and on radiographs, instead of CT-scans. The alignment measurements were also made on radiographs, instead of CT-scans.

## 5. Conclusions

This study showed that PSI-guided double chevron-cut close-wedge DFO followed by immediate weight-bearing as tolerated is accurate, safe, and effective in the correction of genu valgum deformity and the associated lateral compartment osteoarthritis.

## Figures and Tables

**Table 1:** Demographic data of patients.

Characteristic	Value
Sex (men:women ratio)	3:20
Age (years, mean and range)	64 (47–80)
Involved extremity (Right:Left)	15:11
BMI (mean ± SD and range)	27 ± 5 (19–37)
Smoking	3
Kellgren–Lawrence grade	
1	12
2	11
3	2
Follow up (months, mean ± SD and range)	17 ± 8 (12–37)

BMI = Body mass index.

**Table 2:** Intraoperative and perioperative parameters.

	Value
Operative time (minutes)	58.8 ± 18.3
Intraoperative radiograph (number)	6.2 ± 1.3
Preoperative Hb (g/dL)	13.5 ± 1.3
Hb level on postoperative day 1 (g/dL)	12.1 ± 1.2

Values presented in mean ± standard deviation; Hb = hemoglobin.

**Table 3:** Clinical and patient-rated outcomes before and after operation.

Outcomes	Values		
	Pre-Operation	Post-Operation	*p*-Value
Active ROM (°)	131 ± 4	128 ± 10	0.18
OKS score	27.6 ± 11.7	39.1 ± 7.5	0.03 *
KSS score	92.1 ± 13.0	143.9 ± 10.2	<0.001 *

Values presented in mean ± standard deviation; ROM = range of motion, OKS = Oxford Knee Score, KSS = 2011 Knee Society Score; * Statistically significant.

**Table 4:** Radiological results before and after operation.

Outcomes	Values		
	Pre-Operation	2 Days Post-Operation (*p* Value)	Final (*p* Value)
WBL ratio (%)	78.7 ± 12.0	48.7 ± 2.9 (<0.001) *	48.1 ± 3.8 (0.44)
HKA (°)	9.3 ± 2.8	−0.5 ± 1.1 (<0.001) *	−0.3 ± 1.4 (0.58)
LDFA (°)	83.6 ± 1.9	91.4 ± 3.5 (<0.001) *	90.8 ± 3.7 (0.32)

Values presented in mean ± standard deviation; WBL = weight-bearing line, HKA = hip–knee–ankle angle, LDFA = lateral distal femur angle; * Statistically significant.

## Data Availability

The data presented in this study are available in this article. Further datasets of this study are available from the corresponding author on reasonable request.
